# A Good Friday Concert at the Edinburgh Castle

**Published:** 1889-05-04

**Authors:** 


### A Good Friday Concert at The Edinburgh Castle.

Of late, public attention has been roused to the claims of the vast population swarming in the East End of London. The good cause has enlisted many earnest workers—Besant with his books; the University men at the Toynbee Hall; the People's Palace for recreation and technical education; and the untiring efforts of people like the Rev. Mr. Barnett, Vicar of St. Jude's, Captain Hamilton, Mr. Charrington and others. But of all the names, perhaps, the best known is that of Dr. Barnardo. Some score of years ago the worthy doctor conceived the idea of trying to save a few of the waifs and strays of the London streets, and ever since he has carried on that work with results that can be best described by the epithet "splendid." The passenger from Fenchurch-street Station may see close to the line some large and substantial blocks of houses that are "homes" for the rescued boys. But it is not the present intention to describe the achievements of Dr. Barnardo: that is a volume written in many hundred grateful hearts. Our immediate concern is with one particular feat, the conversion of the "Edinburgh Castle" and the concert of sacred music given there on Good Friday.

Limehouse is a vast district round the docks beyond Stepney, and is best reached by train from Fenchurch-street to Burdett-road. On all sides the visitor sees interminable rows of small houses, with here and there a public-house or a church. The population is dense, and the number of children simply amazing. Years ago there existed right in the heart of crowded Limehouse a music hall of the worst type, attached to a notorious gin-palace, known as The Edinburgh Castle. This place was a source of constant trouble to the police, and a nuisance to all the respectable inhabitants of the neighbourhood until Dr. Barnardo became its proprietor. The public-house portion retains its old name, and is brilliantly lighted as ever, but coffee is substituted for gin. In the hall itself no less a change has been effected by the magic of the Doctor's touch. The whole has been reconstructed with an especial view to sound, and there is accommodation for about 3,000 persons. In shape it is square, with sloping seats along the sides, and a gallery at the lower end. As one entered the large and brilliantly-lighted room the other evening, the first sensation was one of thorough comfort and homeliness. The walls are panelled with wood, painted in soft warm colours, relieved by a few texts on a red background, fringed with evergreen. Overhead are a couple of hundred electric lamps, the light from which is mellowed by groups of gas lamps scattered about the body of the building. On the platform stands Dr. Barnardo, a pleasant, thoughtful-looking man of about forty years of age. He is somewhat short and wears spectacles, through which he beams on all sides as he opens the meeting with a few sentences in a clear, cheerful voice.

The performance is begun with a solo on the organ—a handsome instrument, standing in one corner of the building, and which has a delightful tone. Then comes a chorus from "The Messiah," given by the Edinburgh Castle Choral Union. There are rather over a hundred performers in the choir, and they sit directly below the platform, facing the audience. At the back is a double row of young men, fringing the lines of girls and women of various ages who supply the female voices. The organist accompanies; but a great feature is the presence of a cornet, which does yeoman service. The player is well out of sight, and can be seen only by a few of those nearest to the orchestra. By his help weak passages are strengthened, and the proper pitch—always a difficult thing in choruses—is maintained throughout. The choir has been well drilled, and their efforts are thoroughly appreciated by the audience, which may be roughly estimated as numbering about two thousand. As each one pays a penny for admission, besides a few reserved seats at sixpence, it is fair to assume that the expenses of the hall for the evening will be covered, a fact which speaks volumes for the appreciation of sacred music at the East End. The next item on the programme is a bass solo by Signor Villa, whose fine voice can be heard to advantage in the large hall. This is followed by a song from Miss Julia Jones, evidently a great favourite. Enquiry showed that this lady is an old friend, and often sings at the Sunday and other services, which to a slight extent explains the enthusiastic encore given to her artistic work. Another chorus followed from the choir and then Miss Agnes Janson sings Barri's "Gethsemane," being followed by Mr. Claude Ravenhill, who renders a solo in a superb tenor voice. During the interval several announcements were made by Dr. Barnardo as to meetings of various kinds, including an assault-at-arms, for which an early application for tickets is advised. The second part was opened by a pianoforte solo by Miss Eva Lonsdale, who has played the singers' accompaniments. The lady in question interpreted a march of Chopin in good style, notwithstanding the difficulty of filling so large a space with sound from an "upright" piano. The masterly work of the pianiste kept up the high professional tone of the concert generally. The rest of the programme went off smoothly, with one or two encores. Among the latter Miss Norah Morison looked very pretty and charming as she returned to an enthusiastic recall for Handel's composition "Oh, had I Jubal's lyre."

The result of Dr. Barnardo's bold experiment in music-hall conversion is certainly of a most gratifying nature. The place is now filled with quiet, orderly people of all sexes and ages, who show a deep interest in all that goes on. Low and vulgar songs are replaced by selections from the great classical composers, and these are sung by trained artistes. Music is extolled by many people as a great educational lever, and the excellence of Good Friday's performance in Dr. Barnardo's converted castle seemed to secure the sympathies of the East End audience from beginning to end.

